# Supplementary material for: Early Outpatient Treatment of COVID-19: A Retrospective Analysis of 392 Cases in Italy
Source: J Clin Med. 2022 Oct 18;11(20):6138. doi: 10.3390/jcm11206138 (PMC9605012; doi:10.3390/jcm11206138)
Supplement: Supplementary file 1 [file Supplementary Data.pdf › Supplementary Data.pdf]

Code	Physician	Patient data			Anthropometric data						Chronic diseases							Delay	Stage when at starting care date					Swab	Prescribed therapy										Hospital	Outcome		
ID	Name	Date care started (dd/mm/aaaaa)	Age	Sex	Weight (kg)	Height (m)	Overweight	Obese	BMI	Weight class	Autoimmune	Cardiovascular	Metabolic	Neoplasms	Neurologic	Respiratory	Total	Days elapsed fron initial symptoms to care/therapy start	Stage	Stage 0	Stage 1	Stage 2A	Stage 2B	tested (pos/neg)/not tested	Suppl./vitamines	FANS	Antib.	HCQ	Colchicine	Ivermect.	Stage 1 drugs	Enoxap./Fond	Corticosteroids	O2	Hospitalization	Recovered	Sequelae	Deceased
1	Allasino	07/03/2021	33	M	miss	miss	miss	miss	miss	miss	no	no	no	no	no		0	2	1		x			tested (pos)	yes	yes	yes	yes	no	no	3	no	yes	no	no	yes	no	no
2	Allasino	07/03/2021	30	F	miss	miss	miss	miss	miss	miss	no	no	no	no	no		0	7	2A			x		tested (pos)	yes	yes	no	no	no	no	1	no	no	no	no	yes	no	no
3	Allasino	07/03/2021	4	F	17	miss	miss	miss	miss	miss	no	no	no	no	no		0	3	1		x			tested (pos)	yes	no	no	no	no	no	0	no	no	no	no	yes	no	no
4	Allasino	07/03/2021	2	F	13	miss	miss	miss	miss	miss	no	no	no	no	no		0	3	1		x			tested (pos)	yes	yes	no	no	no	no	1	no	no	no	no	yes	no	no
5	Allasino	03/03/2021	57	F	miss	miss	miss	miss	miss	miss	no	no	no	no	no		0	4	2B				x	tested (pos)	yes	yes	yes	no	no	no	2	no	yes	no	no	yes	no	no
6	Allasino	03/03/2021	57	M	miss	miss	miss	miss	miss	miss	no	no	no	no	no		0	3	2A			x		tested (pos)	yes	yes	yes	no	no	no	2	no	no	no	no	yes	no	no
7	Allasino	05/03/2021	57	F	miss	miss	miss	miss	miss	miss	no	no	no	no	no		0	10	2A			x		tested (pos)	yes	yes	yes	no	no	no	2	no	yes	no	no	yes	no	no
8	Allasino	06/03/2021	50	F	70	1.72			23.66	Normal weight	no	no	yes	no	no		1	5	1		x			tested (pos)	yes	yes	no	no	no	no	1	no	no	no	no	yes	no	no
9	Allasino	06/03/2021	21	F	58	1.73	no	no	19.38	Normal weight	no	no	no	no	no		0	5	1		x			tested (pos)	yes	yes	yes	no	no	no	2	no	no	no	no	yes	no	no
10	Allasino	10/03/2021	55	M	89	1.78	yes	no	28.09	Overweight	no	no	yes	no	no		1	9	2B				x	tested (pos)	yes	yes	yes	yes	no	no	3	no	yes	yes	no	yes	no	no
11	Allasino	05/03/2021	69	F	miss	miss	no	no	miss	miss	no	yes	no	no	no		1	4	2A			x		tested (pos)	yes	yes	yes	no	no	no	2	yes	yes	no	yes	yes	yes	no
13	Allasino	19/02/2021	40	F	miss	miss	yes	yes	miss	miss	no	yes	no	no	no		1	3	2A			x		tested (pos)	yes	yes	yes	no	no	no	2	no	no	no	no	yes	no	no
14	Allasino	19/02/2021	43	M	miss	miss	no	no	miss	miss	no	no	no	no	no		0	3	1		x			tested (pos)	yes	yes	no	no	no	no	1	no	no	no	no	yes	no	no
15	Allasino	28/03/2021	65	F	miss	miss	miss	miss	miss	miss	no	no	no	no	no		0	3	2A			x		tested (pos)	yes	yes	yes	no	no	no	2	no	yes	no	no	yes	no	no
16	Allasino	27/03/2021	50	F	68	1.7	no	no	23.53	Normal weight	no	no	no	no	no		0	3	2A			x		tested (pos)	yes	yes	yes	no	no	no	2	no	no	no	no	yes	no	no
17	Allasino	17/03/2021	70	F	miss	miss	miss	miss	miss	miss	no	no	no	no	no		0	9	1		x			tested (pos)	yes	yes	no	no	no	no	1	no	no	no	no	yes	no	no
18	Allasino	20/02/2021	44	F	miss	miss	no	no	miss	miss	no	no	yes	no	no		1	3	1		x			tested (pos)	yes	yes	no	no	no	no	1	no	no	no	no	yes	no	no
19	Allasino	21/02/2021	miss	M	miss	miss	miss	miss	miss	miss	no	yes	no	no	no		1	4	2A			x		tested (pos)	yes	yes	yes	no	no	no	2	no	no	no	no	yes	no	no
20	Allasino	21/02/2021	miss	F	miss	miss	miss	miss	miss	miss	no	no	no	no	no		0	5	2A			x		tested (pos)	yes	yes	yes	yes	no	no	3	no	yes	no	no	yes	no	no
21	Allasino	27/02/2021	35	M	65	1.75	no	no	21.22	Normal weight	no	no	no	no	no		0	3	1		x			tested (pos)	yes	yes	yes	no	no	no	2	no	no	no	no	yes	no	no
22	Allasino	27/02/2021	33	F	56	1.7	no	no	19.38	Normal weight	no	no	no	no	no		0	3	2A			x		tested (pos)	yes	yes	yes	no	no	no	2	no	yes	no	no	yes	no	no
23	Allasino	05/03/2021	59	M	miss	miss	miss	miss	miss	miss	no	no	no	no	no		0	1	2A			x		tested (pos)	yes	yes	no	no	no	no	1	no	no	no	no	yes	no	no
24	Allasino	05/03/2021	53	F	miss	miss	miss	miss	miss	miss	no	no	no	no	no		0	1	2A			x		tested (pos)	yes	yes	yes	no	no	no	2	no	yes	no	no	yes	no	no
25	Allasino	05/03/2021	22	F	miss	miss	miss	miss	miss	miss	no	no	no	no	no		0	[illegible]	[illegible]	[illegible]	[illegible]	[illegible]	[illegible]	[illegible]	[illegible]	[illegible]	[illegible]	[illegible]	[illegible]	[illegible]	[illegible]	[illegible]	[illegible]	[illegible]	[illegible]	[illegible]	[illegible]	[illegible]
[illegible]	[illegible]	[illegible]	[illegible]	[illegible]	[illegible]	[illegible]	[illegible]	[illegible]	[illegible]	[illegible]	[illegible]	[illegible]	[illegible]	[illegible]	[illegible]	[illegible]	[illegible]	[illegible]	[illegible]	[illegible]	[illegible]	[illegible]	[illegible]	[illegible]	[illegible]	[illegible]	[illegible]	[illegible]	[illegible]	[illegible]	[illegible]	[illegible]	[illegible]	[illegible]	[illegible]	[illegible]	[illegible]	[illegible]
