# Supplementary material for: Early Outpatient Treatment of COVID-19: A Retrospective Analysis of 392 Cases in Italy
Source: J Clin Med. 2022 Oct 18;11(20):6138. doi: 10.3390/jcm11206138 (PMC9605012; doi:10.3390/jcm11206138)
Supplement: Supplementary file 1 [file jcm-11-06138-s001.zip › Supplementary Tables.pdf]

Supplementary Table S1. Vitamins and supplements recommended in COVID-19 phase 0 (asymptomatic with positive swab).

Drug	Dosage	Contraindications/warnings	Selected references
vitamin D3	50,000 IU/die for 6 days (if not already taking as prophylaxis) or 10,000 IU/day for 6 days (if already taking as prophylaxis). Continue thereafter with 4,000 IU/day with a high-fat meal (i.e. at lunch). In children: 200 IU/Kg/day until a negative swab is obtained	- severe chronic renal failure<br>- hypercalcemia	Bae & Kim, 2020; Grant et al., 2020; Xu et al., 2020; https://c19vitamind.com/ [1–4]
vitamin C	minimum 1 g/day, depending on formulation	- severe chronic renal failure<br>- G6PD deficiency	Bae & Kim, 2020; Colunga Biancatelli et al., 2020; Holford et al., 2020, https://c19vitaminc.com/ [1,5–7]
zinc picolinate	30-50 mg/day	- none	Skalny et al., 2020; Wessels et al., 2020; https://c19zinc.com/ [8–10]
hesperidin	100 mg/day	- none	Adhikari et al., 2021; Bellavite & Donzelli, 2020 [11,12]

quercetin	up to 250 mg twice a day	- none	Colunga Biancatelli et al., 2020; Derosa et al., 2021 https://c19quercetin.com/ [6,13,14]
lactoferrin	up to 200 mg twice a day	- none	Chang et al., 2020; Wang et al., 2020 [15,16]
bromhexine	8 mg three times a day.	- none	Depfenhart et al., 2020 [17]
Pelargonium sidoides	6 to 12 years of age: 13 mg 3 times a day for 7 days. Over 12 years of age: 20diatesi mg 3 times a day for 7 days	- haemorrhagic diathesis (for example, treatment with anticoagulants)	Brendler et al., 2021 [18]
fumigations	3 times a day	- none	
hydroxytyrosol and α-cyclodextrin	2-3 oropharyngeal sprays 3-4 times a day	- none	Carrouel et al., 2021; Kiani et al., 2020 [19,20]
vitamin A	up to 30,000 IU/day	- pregnancy	Midha et al., 2021; Trasino, 2020 [21,22]
resveratrol	up to 1,000 mg/day		Filardo et al., 2020 [23]

Important note: doctor will choose among the various options according to the profile and needs of individual patients. As a general recommendation, vitamin D3, vitamin C and zinc picolinate should be always included in any plan, also in more advanced phases of COVID-19.

Supplementary Table S2. Drugs recommended in COVID-19 phase 1 (symptomatic without signs of lung disease).

**Drug**	**Dosage**	**Contraindications/warnings**	**Selected references**
acetylsalicylic acid	100 mg/day until symptoms subside		Bianconi et al., 2020; Cacciapuoti & Cacciapuoti, 2021; https://c19aspirin.com/ [24–26]
Ivermectin	0.2 mg/kg up to 20 mg total dose. If insufficient clinical response, severe disease or obesity, daily dose can be increased up to 0.4-0.6 mg/kg		Kory et al., 2021; Marik & Kory, 2021; https://c19ivermectin.com/ [27–29]
Hydroxychloroquine	200 mg twice a day for 7 days	- arrhythmia<br>- retinopathy<br>- G6PD deficiency	Million et al., 2021; Nina & Dash, 2020; Sinha & Balayla, 2020; https://c19hcq.com/ [30–33]
Azithromycin	500 mg/day for 3 days, stop for 2 days, then repeat	- long Q-T Syndrome	Million et al., 2021; Echeverría-Esnal et al., 2021; [30,34]
doxycycline (as an alternative to azithromycin)	100 mg twice a day for 7 days		Yates et al., 2020 [35]
Colchicine	0.5 mg twice a day for 14 days	- cardiac insufficiency<br>- severe chronic renal failure	Reyes et al., 2021; https://c19colchicine.com/ [36,37]

Important note: ivermectin, hydroxychloroquine and colchicine can be prescribed only with the patient's prior written informed consent, as required by Italian law (art. 3 paragraph 2 of the Law n. 94/98, published in the Official Gazette no. 86, 14 April 1998).

Supplementary Table S3. Drugs recommended in COVID-19 phase 2a (symptomatic with lung disease).

**Drug**	**Dosage**	**Contraindications/warnings**	**Selected references**
amoxicillin/clavulanic acid (consider associating azithromycin in case of bacterial superinfection)	875 mg+125 mg 3 times a day for 8-10 days		
Enoxaparin	4.000 IU/day for 10 days if <90 Kg bw<br>6.000 IU/day for 10 days if >90 Kg bw	- haemorrhagic diathesis<br>- thrombocytopaenia	Drago et al., 2020; Susen et al., 2020 [38,39]
Levodropropizine	60 mg as needed, up to 3 times a day		
Acetylcysteine	600 mg up to 3 times a day for 7 days		Shi & Puyo, 2020 [40]
Montelukast	10 mg 2 hours after dinner for 14 days		Aigner et al., 2020 [41]

Important note: doctors may consider already in this phase glucocorticoids recommended in COVID-19 phase 2b.

Supplementary Table S4. Drugs recommended in COVID-19 phase 2b (symptomatic with lung disease and desaturation).

**Therapy**	**Dosage**	**Contraindications/warnings**	**Selected references**
oxygen therapy	1-6 L/min if SpO_2 <92% in ambient air (*)		
Enoxaparin	100 IU/Kg/12 hours		Drago et al., 2020; Susen et al., 2020 [38,39]
Glucocorticoids			Alexaki and Henneicke, 2021 [42]
Dexamethasone	6 mg every morning or 3 mg twice a day	- do not associate, use as alternatives<br>- use gastroprotection<br>- carefully monitor for possible hyperglycemia, elevated blood pressure, and other common glucocorticoid-associated adverse effects	
Betamethasone	8 mg every morning or 4 mg twice a day		
Methylprednisolone	32 mg every morning or 16 mg twice a day		
Prednisone	40 mg every morning or 20 mg twice a day		
Deflazacort	30 mg twice a day		
Antibiotics	according to clinical judgement		

Important note: never use glucocorticoids at the beginning symptoms, wait until the end of the viremic phase. Treatment should be up to 6-7 days, thereafter taper off the drug. Prefer a single administration in the morning, consider two administrations in suffering patients. Deflazacort has a short half-life and should be always given in two separate administrations.

(*) hospitalization is mandatory whenever >6 L/min needed and/or SpO_2 permanently < 92%.

References

1. Bae, M.; Kim, H. Mini-Review on the Roles of Vitamin C, Vitamin D, and Selenium in the Immune System against COVID-19. *Molecules* **2020**, *25*, E5346, doi:10.3390/molecules25225346.
2. Grant, W.B.; Lahore, H.; McDonnell, S.L.; Baggerly, C.A.; French, C.B.; Aliano, J.L.; Bhattoa, H.P. Evidence That Vitamin D Supplementation Could Reduce Risk of Influenza and COVID-19 Infections and Deaths. *Nutrients* **2020**, *12*, E988, doi:10.3390/nu12040988.
3. Xu, Y.; Baylink, D.J.; Chen, C.-S.; Reeves, M.E.; Xiao, J.; Lacy, C.; Lau, E.; Cao, H. The Importance of Vitamin d Metabolism as a Potential Prophylactic, Immunoregulatory and Neuroprotective Treatment for COVID-19. *J Transl Med* **2020**, *18*, 322, doi:10.1186/s12967-020-02488-5.
4. Vitamin D for COVID-19: Real-Time Analysis of All 315 Studies Available online: https://c19vitamind.com/ (accessed on 17 October 2022).
5. Holford, P.; Carr, A.C.; Jovic, T.H.; Ali, S.R.; Whitaker, I.S.; Marik, P.E.; Smith, A.D. Vitamin C—An Adjunctive Therapy for Respiratory Infection, Sepsis and COVID-19. *Nutrients* **2020**, *12*, 3760, doi:10.3390/nu12123760.
6. Colunga Biancatelli, R.M.L.; Berrill, M.; Catravas, J.D.; Marik, P.E. Quercetin and Vitamin C: An Experimental, Synergistic Therapy for the Prevention and Treatment of SARS-CoV-2 Related Disease (COVID-19). *Front Immunol* **2020**, *11*, 1451, doi:10.3389/fimmu.2020.01451.
7. Vitamin C for COVID-19: Real-Time Analysis of All 86 Studies Available online: https://c19early.com/ (accessed on 17 October 2022).
8. Wessels, I.; Rolles, B.; Rink, L. The Potential Impact of Zinc Supplementation on COVID-19 Pathogenesis. *Front Immunol* **2020**, *11*, 1712, doi:10.3389/fimmu.2020.01712.
9. Skalny, A.V.; Rink, L.; Ajsuvakova, O.P.; Aschner, M.; Gritsenko, V.A.; Alekseenko, S.I.; Svistunov, A.A.; Petrakis, D.; Spandidos, D.A.; Aaseth, J.; et al. Zinc and Respiratory Tract Infections: Perspectives for COVID-19 (Review). *Int J Mol Med* **2020**, *46*, 17–26, doi:10.3892/ijmm.2020.4575.
10. Zinc for COVID-19: Real-Time Analysis of All 90 Studies Available online: https://c19zinc.com/ (accessed on 17 October 2022).
11. Bellavite, P.; Donzelli, A. Hesperidin and SARS-CoV-2: New Light on the Healthy Function of Citrus Fruits. *Antioxidants (Basel)* **2020**, *9*, 742, doi:10.3390/antiox9080742.
12. Adhikari, B.; Marasini, B.P.; Rayamajhee, B.; Bhattarai, B.R.; Lamichhane, G.; Khadayat, K.; Adhikari, A.; Khanal, S.; Parajuli, N. Potential Roles of Medicinal Plants for the Treatment of Viral Diseases Focusing on COVID-19: A Review. *Phytother Res* **2021**, *35*, 1298–1312, doi:10.1002/ptr.6893.
13. Derosa, G.; Maffioli, P.; D'Angelo, A.; Di Pierro, F. A Role for Quercetin in Coronavirus Disease 2019 (COVID-19). *Phytother Res* **2021**, *35*, 1230–1236, doi:10.1002/ptr.6887.
14. Quercetin for COVID-19: Real-Time Analysis of All 22 Studies Available online: https://c19quercetin.com/ (accessed on 17 October 2022).
15. Wang, Y.; Wang, P.; Wang, H.; Luo, Y.; Wan, L.; Jiang, M.; Chu, Y. Lactoferrin for the Treatment of COVID-19 (Review). *Exp Ther Med* **2020**, *20*, 272, doi:10.3892/etm.2020.9402.
16. Chang, R.; Ng, T.B.; Sun, W.-Z. Lactoferrin as Potential Preventative and Adjunct Treatment for COVID-19. *Int J Antimicrob Agents* **2020**, *56*, 106118, doi:10.1016/j.ijantimicag.2020.106118.
17. Depfenhart, M.; de Villiers, D.; Lemperle, G.; Meyer, M.; Di Somma, S. Potential New Treatment Strategies for COVID-19: Is There a Role for Bromhexine as Add-on Therapy? *Intern Emerg Med* **2020**, *15*, 801–812, doi:10.1007/s11739-020-02383-3.

18. Brendler, T.; Al-Harrasi, A.; Bauer, R.; Gafner, S.; Hardy, M.L.; Heinrich, M.; Hosseinzadeh, H.; Izzo, A.A.; Michaelis, M.; Nassiri-Asl, M.; et al. Botanical Drugs and Supplements Affecting the Immune Response in the Time of COVID-19: Implications for Research and Clinical Practice. *Phytother Res* **2021**, *35*, 3013–3031, doi:10.1002/ptr.7008.
19. Kiani, A.K.; Dhuli, K.; Anpilogov, K.; Bressan, S.; Dautaj, A.; Dundar, M.; Beccari, T.; Ergoren, M.C.; Bertelli, M. Natural Compounds as Inhibitors of SARS-CoV-2 Endocytosis: A Promising Approach against COVID-19. *Acta Biomed* **2020**, *91*, e2020008, doi:10.23750/abm.v91i13-S.10520.
20. Carrouel, F.; Gonçalves, L.S.; Conte, M.P.; Campus, G.; Fisher, J.; Fraticelli, L.; Gadea-Deschamps, E.; Ottolenghi, L.; Bourgeois, D. Antiviral Activity of Reagents in Mouth Rinses against SARS-CoV-2. *J Dent Res* **2021**, *100*, 124–132, doi:10.1177/0022034520967933.
21. Trasino, S.E. A Role for Retinoids in the Treatment of COVID-19? *Clin Exp Pharmacol Physiol* **2020**, *47*, 1765–1767, doi:10.1111/1440-1681.13354.
22. Midha, I.K.; Kumar, N.; Kumar, A.; Madan, T. Mega Doses of Retinol: A Possible Immunomodulation in Covid-19 Illness in Resource-Limited Settings. *Rev Med Virol* **2021**, *31*, 1–14, doi:10.1002/rmv.2204.
23. Filardo, S.; Di Pietro, M.; Mastromarino, P.; Sessa, R. Therapeutic Potential of Resveratrol against Emerging Respiratory Viral Infections. *Pharmacol Ther* **2020**, *214*, 107613, doi:10.1016/j.pharmthera.2020.107613.
24. Bianconi, V.; Violi, F.; Fallarino, F.; Pignatelli, P.; Sahebkar, A.; Pirro, M. Is Acetylsalicylic Acid a Safe and Potentially Useful Choice for Adult Patients with COVID-19 ? *Drugs* **2020**, *80*, 1383–1396, doi:10.1007/s40265-020-01365-1.
25. Cacciapuoti, F.; Cacciapuoti, F. Could Low Doses Acetylsalicylic Acid Prevent Thrombotic Complications in COVID-19 Patients? *Clin Appl Thromb Hemost* **2021**, *27*, 10760296211014592, doi:10.1177/10760296211014592.
26. Aspirin for COVID-19: Real-Time Analysis of All 61 Studies Available online: https://c19aspirin.com/ (accessed on 17 October 2022).
27. Kory, P.; Meduri, G.U.; Varon, J.; Iglesias, J.; Marik, P.E. Review of the Emerging Evidence Demonstrating the Efficacy of Ivermectin in the Prophylaxis and Treatment of COVID-19. *Am J Ther* **2021**, *28*, e299–e318, doi:10.1097/MJT.0000000000001377.
28. Marik, P.E.; Kory, P. Ivermectin, A Reanalysis of the Data. *Am J Ther* **2021**, *28*, e579–e580, doi:10.1097/MJT.0000000000001443.
29. Ivermectin for COVID-19: Real-Time Analysis of All 185 Studies Available online: https://c19ivermectin.com/ (accessed on 17 October 2022).
30. Million, M.; Roussel, Y.; Gautret, P.; Raoult, D. Effect of Hydroxychloroquine and Azithromycin on SARS-CoV-2 Clearance in COVID-19 Patients, a Meta-Analysis. *Int J Antimicrob Agents* **2021**, *57*, 106240, doi:10.1016/j.ijantimicag.2020.106240.
31. Nina, P.B.; Dash, A.P. Hydroxychloroquine as Prophylaxis or Treatment for COVID-19: What Does the Evidence Say? *Indian J Public Health* **2020**, *64*, S125–S127, doi:10.4103/ijph.IJPH_496_20.
32. Sinha, N.; Balayla, G. Hydroxychloroquine and COVID-19. *Postgrad Med J* **2020**, *96*, 550–555, doi:10.1136/postgradmedj-2020-137785.
33. HCQ for COVID-19: Real-Time Analysis of All 444 Studies Available online: https://c19hcq.com/ (accessed on 17 October 2022).
34. Echeverría-Esnal, D.; Martin-Ontiyuelo, C.; Navarrete-Rouco, M.E.; De-Antonio Cuscó, M.; Ferrández, O.; Horcajada, J.P.; Grau, S. Azithromycin in the Treatment of COVID-19: A Review. *Expert Rev Anti Infect Ther* **2021**, *19*, 147–163, doi:10.1080/14787210.2020.1813024.
35. Yates, P.A.; Newman, S.A.; Oshry, L.J.; Glassman, R.H.; Leone, A.M.; Reichel, E. Doxycycline Treatment of High-Risk COVID-19-Positive Patients with Comorbid Pulmonary Disease. *Ther Adv Respir Dis* **2020**, *14*, 1753466620951053, doi:10.1177/1753466620951053.

36. Reyes, A.Z.; Hu, K.A.; Teperman, J.; Wampler Muskardin, T.L.; Tardif, J.-C.; Shah, B.; Pillinger, M.H. Anti-Inflammatory Therapy for COVID-19 Infection: The Case for Colchicine. *Ann Rheum Dis* **2021**, *80*, 550–557, doi:10.1136/annrheumdis-2020-219174.
37. Colchicine for COVID-19: Real-Time Analysis of All 39 Studies Available online: https://c19colchicine.com/ (accessed on 17 October 2022).
38. Drago, F.; Gozzo, L.; Li, L.; Stella, A.; Cosmi, B. Use of Enoxaparin to Counteract COVID-19 Infection and Reduce Thromboembolic Venous Complications: A Review of the Current Evidence. *Front Pharmacol* **2020**, *11*, 579886, doi:10.3389/fphar.2020.579886.
39. Susen, S.; Tacquard, C.A.; Godon, A.; Mansour, A.; Garrigue, D.; Nguyen, P.; Godier, A.; Testa, S.; Levy, J.H.; Albaladejo, P.; et al. Prevention of Thrombotic Risk in Hospitalized Patients with COVID-19 and Hemostasis Monitoring. *Crit Care* **2020**, *24*, 364, doi:10.1186/s13054-020-03000-7.
40. Shi, Z.; Puyo, C.A. N-Acetylcysteine to Combat COVID-19: An Evidence Review. *Ther Clin Risk Manag* **2020**, *16*, 1047–1055, doi:10.2147/TCRM.S273700.
41. Aigner, L.; Pietrantonio, F.; Bessa de Sousa, D.M.; Michael, J.; Schuster, D.; Reitsamer, H.A.; Zerbe, H.; Studnicka, M. The Leukotriene Receptor Antagonist Montelukast as a Potential COVID-19 Therapeutic. *Front Mol Biosci* **2020**, *7*, 610132, doi:10.3389/fmolb.2020.610132.
42. Alexaki, V.I.; Henneicke, H. The Role of Glucocorticoids in the Management of COVID-19. *Horm Metab Res* **2021**, *53*, 9–15, doi:10.1055/a-1300-2550.
