# Supplementary material for: Early Outpatient Treatment of COVID-19: A Retrospective Analysis of 392 Cases in Italy
Source: J Clin Med. 2022 Oct 18;11(20):6138. doi: 10.3390/jcm11206138 (PMC9605012; doi:10.3390/jcm11206138)
Supplement: Supplementary file 1 [file jcm-11-06138-s001.zip › Supplementary Material Questionnaire for Physicians.pdf]

EARLY OUTPATIENT TREATMENT OF COVID-19: A RETROSPECTIVE ANALYSIS OF 392 CASES IN ITALY

Questionnaire for physicians

Name ____ date _____________________

Answer please to the following questions having in mind your attitudes and experience during the time interval included in the study (1/11/20-31/3/21).

1. How often did you prescribe **vitamins and supplements** in:

	never	sometimes	often	always
**Covid-19 stage 0**				
**Covid-19 stage 1**				
**Covid-19 stage 2a**				
**Covid-19 stage 2b**				

(for each stage, only one choice allowed)

2. How often did you prescribe **aspirin** in:

	never	sometimes	often	always
**Covid-19 stage 0**				
**Covid-19 stage 1**				
**Covid-19 stage 2a**				
**Covid-19 stage 2b**				

(for each stage, only one choice allowed)

2.1 List please criteria supporting your choice (for example presence/absence of any signs/symptoms, risk factors, indications/contraindications)

3. How often did you prescribe **antibiotics** in:

	never	sometimes	often	always
**Covid-19 stage 0**				
**Covid-19 stage 1**				
**Covid-19 stage 2a**				
**Covid-19 stage 2b**				

(for each stage, only one choice allowed)

3.1 List please criteria supporting your choice (for example presence/absence of any signs/symptoms, risk factors, indications/contraindications)

4. How often did you prescribe **hydroxychloroquine** in:

	never	sometimes	often	always
**Covid-19 stage 0**				
**Covid-19 stage 1**				
**Covid-19 stage 2a**				
**Covid-19 stage 2b**				

(for each stage, only one choice allowed)

4.1 List please criteria supporting your choice (for example presence/absence of any signs/symptoms, risk factors, indications/contraindications)

5. How often did you prescribe **ivermectin** in:

	never	sometimes	often	always
**Covid-19 stage 0**				
**Covid-19 stage 1**				
**Covid-19 stage 2a**				
**Covid-19 stage 2b**				

(for each stage, only one choice allowed)

5.1 List please criteria supporting your choice (for example presence/absence of any signs/symptoms, risk factors, indications/contraindications)

6. How often did you prescribe **colchicine** in:

	never	sometimes	often	always
**Covid-19 stage 0**				
**Covid-19 stage 1**				
**Covid-19 stage 2a**				
**Covid-19 stage 2b**				

(for each stage, only one choice allowed)

6.1 List please criteria supporting your choice (for example presence/absence of any signs/symptoms, risk factors, indications/contraindications)

7. How often did you prescribe **enoxaparin** in:

	never	sometimes	often	always
**Covid-19 stage 0**				
**Covid-19 stage 1**				
**Covid-19 stage 2a**				
**Covid-19 stage 2b**				

(for each stage, only one choice allowed)

7.1 List please criteria supporting your choice (for example presence/absence of any signs/symptoms, risk factors, indications/contraindications)

8. How often did you prescribe **glucocorticoids** in:

	never	sometimes	often	always
**Covid-19 stage 0**				
**Covid-19 stage 1**				
**Covid-19 stage 2a**				
**Covid-19 stage 2b**				

(for each stage, only one choice allowed)

8.1 List please criteria supporting your choice (for example presence/absence of any signs/symptoms, risk factors, indications/contraindications)

9. How often did you observe any suspect adverse reactions with each of the following drugs?

	**never**	**uncommonly**	**commonly**	**very commonly**
		<1%	1-10%	>10%
**vitamins and supplements**				
**aspirin**				
**antibiotics**				
**hydroxychloroquine**				
**ivermectin**				
**colchicine**				
**enoxaparin**				
**glucocorticoids**				

10. In case you observed any suspect adverse reactions, please indicate whether they were:

**Grade 1: Mild**<br>*transient or mild discomfort (< 48 hours); no medical intervention or therapy required*	
**Grade 2: Moderate**<br>*mild to moderate limitation in activity; some assistance may be needed; no or minimal medical intervention or therapy required*	
**Grade 3: Severe**<br>*marked limitation in activity, some assistance usually required; medical intervention or therapy required, hospitalizations possible*	
**Grade 4: Life threatening**<br>*extreme limitation in activity, significant assistance required; significant medical intervention or therapy required, hospitalization or hospice care probable*	

11. In case you observed grade 2 or higher suspect adverse reactions, please briefly describe the case(s):
